# Recurrent Implantation Failure—Is It the Egg or the Chicken?

**DOI:** 10.3390/life12010039

**Published:** 2021-12-27

**Authors:** Paul Pirtea, Dominique de Ziegler, Jean Marc Ayoubi

**Affiliations:** Department of Obstetrics and Gyncology and Reproductive Medicine, Hopital Foch, Faculté de Médecine Paris Ouest (UVSQ), 92150 Suresnes, France; dom@deziegler.com (D.d.Z.); jm.ayoubi@hopital-foch.com (J.M.A.)

**Keywords:** reccurent implantation failure, Assisted Reproductive Technique, euploid embryo transfer, frozen embryo transfer

## Abstract

Recurrent implantation failure (RIF) is an undefined, quite often, clinical phenomenon that can result from the repeated failure of embryo transfers to obtain a viable pregnancy. Careful clinical evaluation prior to assisted reproduction can uncover various treatable causes, including endocrine dysfunction, fibroid(s), polyp(s), adhesions, uterine malformations. Despite the fact that it is often encountered and has a critical role in Assisted Reproductive Technique (ART) and human reproduction, RIF’s do not yet have an agreed-on definition, and its etiologic factors have not been entirely determined. ART is a complex treatment with a variable percentage of success among patients and care providers. ART depends on several factors that are not always known and probably not always the same. When confronted with repeated ART failure, medical care providers should try to determine whether the cause is an embryo or endometrium related. One of the most common causes of pregnancy failure is aneuploidy. Therefore, it is likely that this represents a common cause of RIF. Other RIF potential causes include immune and endometrial factors; however, with a very poorly defined role. Recent data indicate that the possible endometrial causes of RIF are very rare, thereby throwing into doubt all endometrial receptivity assays. All recent reports indicate that the true origin of RIF is probably due to the “egg”.

## 1. Introduction

Assisted Reproductive Technique (ART) is a complex treatment with a variable percentage of success among patients and care providers. ART depends on several factors that are not always known and probably not always the same. The human reproduction natural limitations also apply to ART as well. Although we might imagine that controlled multiple ovulation and embryo selection might perform better, it remains limited.

When confronted with repeated ART failure, medical care providers should try to determine whether the cause is embryo or endometrium related, and they must also determine how many ART failures define true repeated implantation failure (RIF) [1].

Despite the increased research and debates regarding RIF, it remains an enigmatic phenomenon with no agreed-on definition. In the current literature, we can find up to 76 variant definitions [2]. The multitude of etiologies constitutes significant medical challenges. Prior to ART, specialized clinical evaluation can often identify multiple treatable causes of RIF, including pathologies like polyps, submucosal myomas, thyroid dysfunction, and/or tobacco consumption. Still, in some cases, more targeted investigations are needed to identify more profound causes.

As ART outcomes improved, so did debates over the definition of RIF multiplied. Despite the increased research regarding this topic, no medical society agreed on a definition and/or minimal measures to follow in the case of RIF. The parameter used for characterizing RIF has been the number of embryos transferred that fail to implant. The early hypothesis proposed that RIF was defined as ART failure after at least 10 embryos were transferred. More recent definitions have proposed the term RIF after the failure to achieve clinical pregnancy with the transfer of four or more good-quality embryos [3] or failure to achieve a clinical pregnancy after the transfer of three or more good-quality embryos [4]. While the majority propose alternate cut-offs, when considering euploidy status and/or maternal age, the definition might be adjusted to two or more failed embryo transfers [5]. Today we can affirm the majority of ART specialists would declare the existence of RIF after at least four failed embryo transfers of good morphology/quality embryos [3].

## 2. RIF: Is it the Egg or the Chicken?

It could be that ART failures do not have the same etiology each time. As mentioned above, it is suggested that the majority of failures are due to aneuploidy [6]. The multitude of reasons that can explain why an embryo fails to implant includes maternal age, low ovarian reserve, suboptimal ART treatment, and poor embryo quality/morphology. 

Embryo implantation is a critical step, and it requires good-quality feminine and masculine gametes. This constitutes a reason to always take into consideration the parental genetic heritage when addressing RIF. Some of the most common genetic anomalies suspected in RIF are translocations found in (1.4% and 3.2%, respectively) of individuals, significantly higher than the rate of translocations reported in couples with infertility (0.3%) [7]. Chromosomal abnormalities were diagnosed in 2.1% of patients (2.5% of females and 1.7% of males), significantly more than what has been reported in women with normal ovarian function (0.6%) [7].

The majority of women diagnosed with RIF probably have aneuploid embryos and/or chromosomally abnormal embryos. Nowadays, with the use of PGT-A, aneuploidy can be diagnosed, and aneuploid embryos can be selected out and excluded from the RIF population. It has been reported by Franasiak et al. that aneuploidy increases after 26 years of age. A slightly increased prevalence was noted at younger ages, with >40% aneuploidy in women 23 years and under. The aneuploidy rate reported was lowest (2% to 6%) in women aged 26 to 37, was 33% at age 42, and was 53% at age 44. Other results provided by PGT-A were 64% involving a single chromosome, 20% two chromosomes, and 16% three chromosomes, with the proportion of more complex aneuploidy increasing with age. Finally, the trisomy/monosomy ratio approximated one and increased minimally with age [8]. Rates of no-euploid transfers have been reported to be 25% in large reports, but these were not broken down by age group [9].

PGT-A is considered by most a great tool in selecting a euploid embryo for transfer. The clinical benefit provided by PGT-A has contesters and has been tested in several randomized clinical trials (RCTs). It is worth mentioning that a recent RCT failed to show a clinical benefit for PGT-A use [10]. Several other authors have reported increased implantation rates [11,12,13,14], reduced time to pregnancy, and decreased miscarriage rates [11,12,13,14]. The American Society of Reproductive Medicine guidelines also mentions that PGT-A may allow for single embryo transfer [15] without compromising pregnancy rates and avoiding multiple gestations complications. 

Although euploid embryos implantation rates are superior to those of non-genetic tested embryos, not all euploid embryos implant, one explanation for this could be the impact of maternal age, even when euploid embryos are transferred. As reported by Franasiak et al., implantation rates of euploid embryos can be variable as early as age 33 years [8]. Another report concerning maternal age by Shapiro et al. also found higher rates of embryo-endometrial asynchrony with increasing maternal age. Their findings show that 50% of embryo transfers were asynchronous in women <35 years old, which is significantly lower compared to 68.1%, which were asynchronous in women >35 years old [16]. Surely, with PGT-A, some embryos are labeled mosaics. In a recent meta-analysis, the mosaic embryo transfers resulted in a significantly lower clinical pregnancy rate (40.1% versus 59.0% versus 48.4%), lower ongoing/live birth rate (27.1% versus 47.0% versus 35.1%), and a higher miscarriage rate (33.3% versus 20.5% versus 27.4%) than euploid and non-PGT transfers, respectively [17]. However, usually care providers transfer them last, and the potential role in RIF when using euploid embryos is not significant.

Apart from a competent embryo and receptive endometrium, implantation requires several microvascular events. It is well established that thrombophilias are linked to multiple obstetric complications, including recurrent pregnancy loss (RPL), and it has now been suggested that it could have a role in implantation failure. Thrombophilia and its impact on RIF are not well understood at this moment. Reports on the mechanism associating RIF to thrombophilias mention an impairment of the initial vascularization induced by a probably limited blood flow to the decidual and/or chorionic vessels [18]. The existing data concerning the aforementioned mechanism are still conflicting and impose validation throw well-designed RCTs. 

Although acquired thrombophilias and antiphospholipid antibodies (aPLs) specifically have a significant association with obstetrical complications and RPL, their implication in RIF has been based on rather conflicting evidence. A 2021 study compared 29 women with RIF medical history with 10 fertile women as a control group, all with measured and compared levels of serum aPLs. The authors found no difference in the aPLs blood levels between the study and control groups, with just one exception. The immunoglobulin G anticardiolipin antibody was found above the normal level in 3% of the RIF group [19]. Other reports suggest the presence of an increased aPLs frequency in women known with RIF, like those present in the case-control study by Sauer et al. [20], where more than one positive aPL was noted in 8% of women with RIF compared with 1.5% in women in the control group, several others did not show an association so far [20].

Antiphospholipid syndrome (APS) criteria require patients to express one clinical criterion and one paraclinical criterion. [21]. Hornstein et al., revealed in a recent meta-analysis that there is no statistically significant association between the clinical pregnancy or ART outcomes and the presence of antiphospholipid antibodies [22]. Other authors reported that women who received APS standard treatment have similar ART results as controls [23]. RIF can be considered as a clinical criterion for antiphospholipid syndrome due to the fact that specific antibodies have been found to be present in both RIF and APS.

## 3. RIF and the Endometrium 

Implantation requires, apart from a quality blastocyst, a selective and receptive endometrium [24]. A suboptimal endometrial receptivity is suspected to be at the origin of 2/3 of implantation failures [25].

The histologic endometrial modifications that follow the variations in hormonal blood levels have been successively studied and quite well characterized [26]. Noyes et al. [27] argued, on a good basis, that on the assessment of secretory histologic modifications in the endometrium allowed for the assignment of endometrial dating. However, the role of endometrial dating as means to improve timing and implantation rates of transferred embryos has not been proven.

Recently, research has been directed towards defining the endometrial modifications during the window of implantation (WOI) and the expression of several important genes in the endometrium. More precisely, some authors suggested the endometrial receptivity analysis (ERA) test by an endometrial biopsy to assess the endometrial receptivity and the progress of endometrial changes [28].

Implantation relies on a cross-talk between the endometrium and the embryo, facilitated by several factors such as growth factors, cytokines, adhesion molecules, and transcription factors. Therefore, we might consider that RIF could be issued due to a modified endometrial receptivity. Modified, advanced or delayed, receptivity might induce modifications in the duration of WOI, which was previously believed to be identical for all women. The regulation and/or dysregulation of several key genes is implicated in the endometrial modifications during the WOI. 

A recent publication by Ruiz-Alonso et al., identified the window of implantation modifications based on 238 genes among women with RIF by making use of the endometrial receptivity array (ERA) [29]. They reported that the window of implantation was modified in 25% of the women and that if the embryo transfer timing was modified according to the data obtained with the use of the ERA biopsy, the implantation rates were similar to those with a normal receptive endometrium WOI. In this study, patients’ mean ages were 38.4 ± 4.7 in the RIF group and 39.1 ± 5.1 in the control group, respectively. However, 34.9% of those in the RIF group and 59.1% of those in the control group obtained embryos issued from oocyte donation. Hashimoto et al. conducted a quite similar study in Japan [30], and with the use of the ERA biopsy test, they identified the endometrium receptivity prior to performing the embryo transfer. These authors did not identify a significant difference in implantation rates between the receptive group and the non-receptive group (32.8% vs. 31.6%, respectively). The actual data linking the ERA biopsy test and RIF is limited to a prospective non-randomized trial and three retrospective studies, all reporting that the frequency of a receptive endometrium could be slightly lower in those women with RIF when compared with controls after benefiting from an ERA biopsy test. These studies report that women with RIF had similar pregnancy rates to those in the control group when the endometrium was determined to be receptive on the ERA biopsy test. Recently, Simon et al., published a prospective study, analyzing the ART results after modifying the embryo transfers timing according to ERA biopsy test results, suggesting a type of personalized embryo transfer (PET) [31]. These authors adjusted the progesterone treatment duration guided by the ERA biopsy test results after decrypting the endometrial transcriptomic profile at the time of WOI [19]. This international study stretched over five years, performed in association with 16 IVF centers, and included in total 266 per-protocol patients divided into three groups, treated with antagonist or agonist controlled ovarian stimulation protocols with or without PGT-A. The study concluded, based on the pregnancy rate, that the personalized embryo transfer was more successful. On the contrary, the same authors reported, miscarriage rates were significantly higher in the PET group and no difference in the live birth rate (LBR) when compared with controls. These results strongly questioned the true value of the PET strategy [19]. In the end, the aforementioned study only strengthens those voices that doubt the efficacy of the ERA biopsy test for embryo transfer timing. The lack of efficacy with the use of PET strategy in concordance with the ERA biopsy test results was also validated by Neves et al. [32], who reported that in their study, ART outcomes were not improved in patients using ERA biopsy test results for PET. Likewise, in good prognosis patients, performing an ERA biopsy test in the cycle prior to the embryo transfer did not show to increase clinical pregnancy rates [32]. In the future, more studies with appropriate control groups and improved methodology are needed in order to validate the clinical relevance of the PET strategy.

Based on the same ideologies, other groups proposed different receptivity tests. It is worth mentioning a receptivity test based on miRNA profiles and the expression of 11 genes selected for their role in the control of endometrial receptivity [32]. Other authors looked into the role of BCL6 expression in the endometrium and the likelihood and or relation of progesterone resistance in women with endometriosis as possible predictors of implantation failure [33]. Recent research showed that in patients with endometriosis, the ART outcomes are similar following frozen euploid blastocyst transfers and therefore validating the hypothesis that any possible detrimental effects endometriosis might exert over the endometrium could be neutralized or bypassed in hormonal substituted cycles used nowadays often for frozen embryo transfers (FET). In patients with unexplained or endometriosis-related infertility, an increased BCL6 expression has identified the endometrium [33]. Although the majority of patients with endometriosis have been found to present an aberrant BCL6 expression, the same may also occur in the absence of evident endometriosis. In any case, increased expression of BCL6, a transcriptional gene repressor, has been related to progesterone resistance by early interfering with progesterone signaling [34]. In patients with unexplained infertility following IVF treatments, a recent study performed by Almquist et al. reported a live birth rate of 11.5% and 58% in patients with and without increased BCL6 expression, respectively [35]. In a prospective non-randomized study, Likes et al., reported that GnRH agonist treatment or endometriotic surgical lesions management could significantly improve ART outcomes [36].

Other authors proposed the search for the ideal immunologic endometrial profile capable of optimizing implantation rates in RIF [37]. Their approach is interesting but not yet clinically validated with an RCT. 

RIF has also been linked with the uterine natural killer cells (uNKS), but the exact role they carry remains still controversial. NK cell level and activity is just one aspect of the immune system involved in women suffering from infertility, and more and better data are needed in order to make this information clinically relevant. Given the menstrual cycles variations also due to hormonal fluctuations makes it extremely hard to measure or establish an immunological profile.

Chronic endometritis (CE) has been diagnosed in several patients with RIF. It is often described with minimal or no clinical symptoms of infection [38]. In the infertile women, Kushnir et al. reported that 45% had CE [39]. The relatively increased prevalence of CF of 14% in RIF patients was confirmed by Bouet et al. [40]. Traditionally CE has been diagnosed using histological examination, or hysteroscopy, and by bacterial culture. Nowadays, the CE diagnosis can be more accurate with the help of Immunohistochemistry CD 138 by identifying the number of stained plasma cells present, although the standard number required for a correct diagnosis has not yet been established. A newer method of CE diagnosis using RT-PCR in order to identify the bacterial DNA with a sensitivity of 75% and a specificity of 100% when compared with the results of other standard tests (hysteroscopy, histology, culture) [41]. The importance of the diagnosis was doubled by the ART outcomes after specific treatment. The implantation rate of those women cured of infection with antibiotics was 37%, compared with 17% in those that were not. The LBR in the IVF cycle after the antibiotic treatment was also 61% compared with 13% in those that did not benefit from antibiotic treatment [38]. It has also been suggested that in certain cases, it is not the chronic inflammation that interferes with implantation rates but rather the modification in the microbiome constituents [41].

Implantation failures could also be a result of several uterine pathologies as myomas, polyps, uterine septum, and intrauterine adhesions, as their prevalence is significant in the RIF population. These abnormalities can impact ART outcomes at times. Very often, these problems are asymptomatic and therefore sometimes underdiagnosed on transvaginal ultrasound, suggesting that other methods of uterine assessment are needed, such as diagnostic hysteroscopy. Several types of myomas can alter uterine contractility and impair several endometrial cytokine expressions. In the case of the uterine septum, women with this pathology have a decreased cumulative pregnancy rate, increased miscarriage rate, increased preterm birth rate, and increased incidence of malpresentation at delivery [42]. The presence of these uterine abnormalities can also disturb normal vascularization as well as induce a mechanical effect on both the endometrium and the myometrium with consequent altered HOXA gene expressions. Surgical treatment of uterine abnormalities can, in some cases, produce complications as intrauterine adhesions and/or infection that consequently can impair implantation.

The female reproductive tract microbiome has been investigated recently, also in relation to RIF. So far, two types of uterine microbial have been correlated to infertility: the *Lactobacillus*-dominated (>90% lactobacilli) and non-*Lactobacillus*-dominated (<90% lactobacilli with >10% of other bacteria). The last one is associated with decreased live birth rates [43]. The microbiome will probably be better understood in the future, but for now, data is still debated given that a different study found no correlation between lactobacilli concentration and pregnancy in ART and reported a dominance of other bacteria, such as *Flavobacterium* spp. [44].

Other add-ons, less used generally, as endometrial scratching or embryo glue have not yet been validated by the existing data.

Although controversial, platelet enriched plasma (PRP) uterine administration has gained a lot of attention recently, and medical providers looked into its use in the case of RIF. Recent findings in a systematic review suggest that PRP is an alternative treatment strategy in patients with thin endometrium and RIF [45]. Given the small amount of good quality data, further prospective, large, and high-quality randomized controlled trials (RCTs) are needed to identify the subpopulation that would most benefit from this PRP treatment.

## 4. RIF and the Embryo

**Figure 1:**
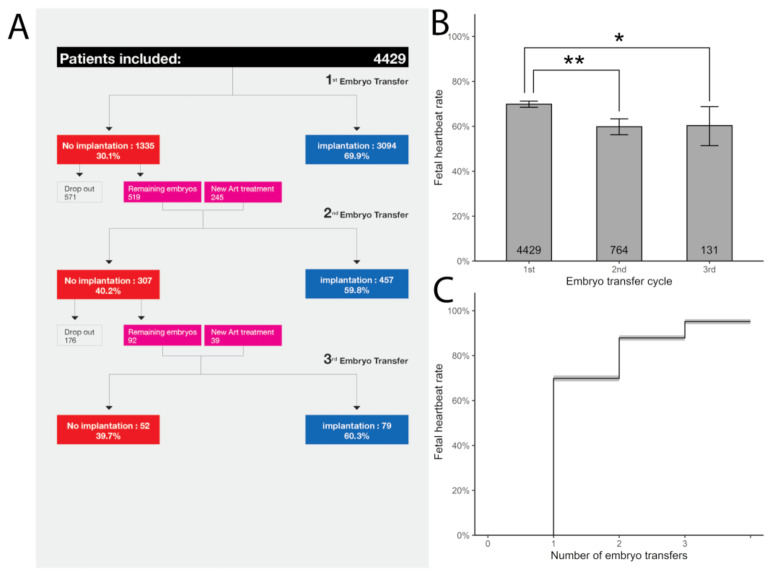
Cumulative implantation rate results after the first, second, and third FE-SET. (**A**) IR was analyzed in women (n = 4429) with up to 3 consecutive FE-SETs. Of those who failed to achieve implantation after the 1st FE-SET (n = 1335), 764 (57.22%) underwent a 2nd FE-SET. Of those who failed to achieve implantation after the 2nd FE- SET (n = 324), 141 (43.51%) patients underwent a 3rd FE-SET. (**B**) Implantation rates after 1st, 2nd, and 3rd cycles. The number of transfers is shown at the bottom of each bar (Tukey’s range test = * *p* = 0.042, ** *p* < 0.001). (**C**) The cumulative IR after up to 3 consecutive FE-SET was 95.2% (95% CI: 94.0%–96.2%) as illustrated by Kaplan-Meier estimates reporting also cumulative IR results after the 1st (69.9%; 95% CI: 68.5%–71.2%), after the 2nd (87.9%; 95% CI: 86.7%–89.0%) FE-SET respectively. The number of transfers is shown at the bottom bar.

Given the uncertainty around the definition for RIF and all the data available, our group considered that the failure of at least three consecutive single euploid frozen blastocyst transfers in women with a normal verified morphological uterus could reasonably characterize the true prevalence of RIF. In order to identify this, our team performed a large retrospective study including a total of 4429 patients that benefited from up to three consecutive single euploid frozen embryo transfers. Our data suggest that the true prevalence of RIF is rare. In patients who are able to obtain euploid blastocysts, 95.2% will achieve a clinical pregnancy after three single euploid embryo transfers. (Figure 1).

**Figure 2:**
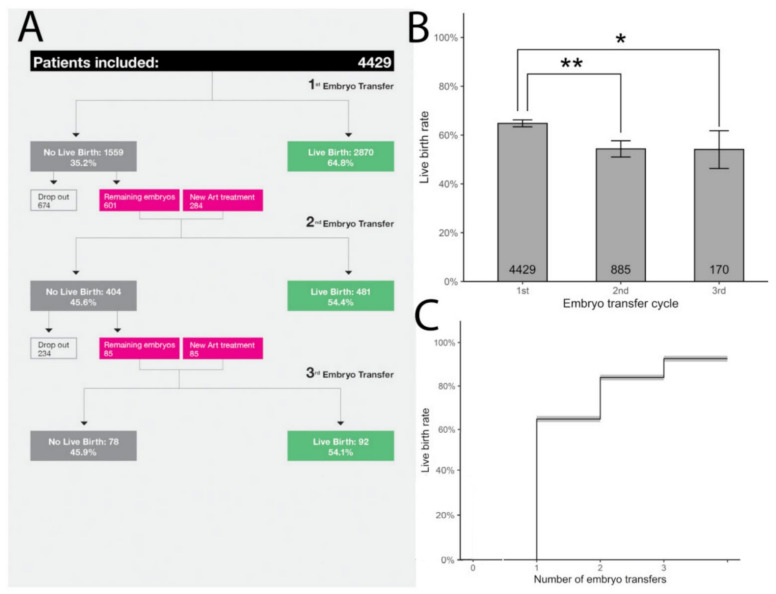
Cumulative live birth rate results after the first, second, and third FE-SET. (**A**) LBR was analyzed in women (n = 4429) with up to 3 consecutive FE-SETs. Of those who failed to achieve LB after the 1st FE-SET (n = 1335), 885 (56.8%) underwent a 2nd FE-SET. Of those who failed to achieve LB after the 2nd FE- SET (n = 404), 170 (42.7%) patients underwent a 3rd FE-SET. (**B**) Live birth rates after 1st, 2nd, and 3rd cycles. The number of transfers is shown at the bottom of each bar (Tukey’s range test = * *p* = 0.01, ** *p* < 0.001). (**C**) The cumulative LBR after up to 3 consecutive FE-SET was 92.6% (95% CI: 91.2%–93.9%) as illustrated by Kaplan-Meier estimates reporting also cumulative LBR after the 1st (64.8%; 95% CI: 63.4%–66.2%), and after the 2nd (83.9%; 95% CI: 82.6%–89.0%) FE-SET respectively. The number of transfers is shown at the bottom bar.

As our data shows, with the increasing embryo transfers, the implantation rates declined minimally. The second and third embryo transfers had a similarly high implantation rate (59.8% vs. 60.3%, respectively) (Figure 1). It is reasonable to estimate that the slight difference between the first, second, and third ETs (69.9% vs. 59.8% and 60.8%) could reflect the morphological embryo-based selection for the transfer order. The constant high implantation rates suggest that successive embryo transfers do not select out women with persistent receptivity abnormalities and that any possible endometrial causes of RIF are very rare. Additionally, less than 5% of those who fail to implant after three euploid embryo transfer attempts may just be unfortunate, and that additional embryo transfers would deliver a good outcome [46]. Additionally, live birth rate (LBR) results after the first, second, and third FE-SET were 64.8%, 54.4%, and 54.1% per transfer, respectively. In patients who are able to obtain euploid blastocysts, 92.6% will achieve a live birth after up to three embryos are transferred (Figure 2).

This large cohort study of repeated FE-SET in women whose uterus was morphologically normal puts serious doubt on the existence of RIF due to persistent endometrial effects. Likewise, the results challenge the soundness of the sometimes-expensive endometrial testing and ensuing therapies that have been broadly deployed for diagnosing and treating RIF of endometrial origin [28,29,47,48] and other various therapies, also named ART add-ons, offered to women who failed to implant following ART treatments. These tests include endometrial immunologic testing [49,50,51], markers for endometriosis induced endometrial alterations [52] and WOI transcriptomic assessment [52,53,54,55,56,57], and personalized transfers. On the contrary, our results suggest that the vast majority of RIFs are of embryonic origin, which can be considerably reduced by transferring embryos tested by PGT-A.

## 5. Conclusion

In conclusion, the endometrial assessments available today may not be useful in treating women with a morphologically normal uterus and RIF.

Actually, based on etiology, RIF seems to be more of an iatrogenic invention than a real diagnosis [58], and thus it produces an opportunity for a lot of unwarranted medical activity. It may be reasonable to simply have the patient undergo another embryo transfer, although the tendency for both patient and clinicians is to want to change something in the face of failure.

These recent data suggest that simply staying the course could provide a very high chance of success and that the true origin of RIF is probably due to the egg. Women that failed one or two embryo transfers cannot be realistically diagnosed with RIF, as they represent probably a normal infertile population, and that success is just delayed. 

## Data Availability

No data reported.
